# Anesthesia management of laparoscopic right colectomy in an older patient with postoperative tetralogy of Fallot with residual anomaly

**DOI:** 10.1186/s40981-024-00707-2

**Published:** 2024-04-11

**Authors:** Satori Mori, Hisakatsu Ito, Sadamu Sugimoto, Daisuke Hibi, Akiyo Kameyama, Masaaki Kawakami, Tomonori Takazawa

**Affiliations:** 1 Department of Anesthesiology, University of Toyama, Sugitani 2630, Toyama, Toyama 930-0194 Japan; 2 Department of Anesthesiology, Kurobe City Hospital, Mikkaiti 1108-1, Kurobe, Toyama 938-8502 Japan

**Keywords:** Tetralogy of Fallot, Laparoscopic surgery, Non-cardiac surgery, Pulmonary to systemic blood flow ratio

## Abstract

**Background:**

Diversity in hemodynamics of adult congenital heart disease necessitates a case-by-case selection of appropriate surgical and anesthetic options. However, previous case reports regarding the management of laparoscopic surgery in adult patients with congenital heart disease are limited.

**Case presentation:**

A 72-year-old man who underwent a laparoscopic right colectomy for colon cancer had a residual ventricular septal defect and right ventricular outflow tract obstruction despite post-repair of tetralogy of Fallot. Pulmonary hypertension or right ventricular dysfunction was not observed. The preoperative pulmonary to systemic blood flow ratio (Qp/Qs) was 2.3. After positive pressure ventilation and insufflation, the amount of left-to-right ventricular shunting decreased, and the Qp/Qs approached 1.0, as calculated from pulmonary arterial and systemic arterial blood gas analysis.

**Conclusions:**

Laparoscopic surgery might be tolerable in patients with tetralogy of Fallot who have preserved the right ventricular function, left-to-right ventricular shunting, and no high pulmonary vascular resistance.

## Background

Treatment advances have prompted an increase in the number of patients with adult congenital heart disease (ACHD) and their percentage has exceeded those of pediatric patients with congenital heart disease (CHD) in 2010 [1]. Tetralogy of Fallot (TOF) is the most common cyanotic congenital heart disease, with an overall incidence approaching 10% of all congenital heart diseases requiring repair surgery [2]. ACHD with cyanosis increases mortality in non-cardiac surgery [1, 3, 4]. Several reports on safe anesthetic management strategies and surgical options for patients with ACHD due to TOF exist.

Herein, we describe a case in which laparoscopic surgery was a good indication for an older patient with residual TOF abnormalities. Hemodynamic changes during anesthesia were numerically captured by measuring the pulmonary-to-systemic blood flow ratio (Qp/Qs) using a pulmonary artery catheter. This report details the advantages of laparoscopic surgery and abdominal insufflation-induced hemodynamic and Qp/Qs changes. In a patient with residual TOF, right ventricular outflow tract obstruction, and ventricular septal defect (VSD) with a left-to-right ventricular shunt, cardiac function was preserved.

## Case presentation

**Fig. 1:**
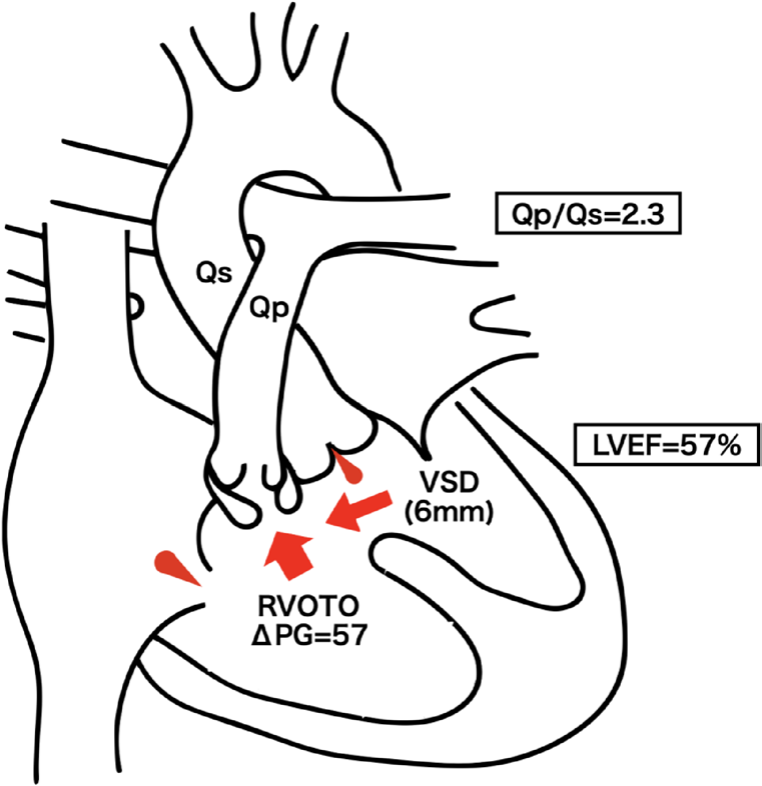
Schema of heart structure in this patient. LVEF, left ventricular ejection fraction; Qp/Qs, pulmonary-to-systemic blood flow ratio; RVOTO, right ventricular outflow tract obstruction; VSD, ventricular septal defect

**Fig. 2:**
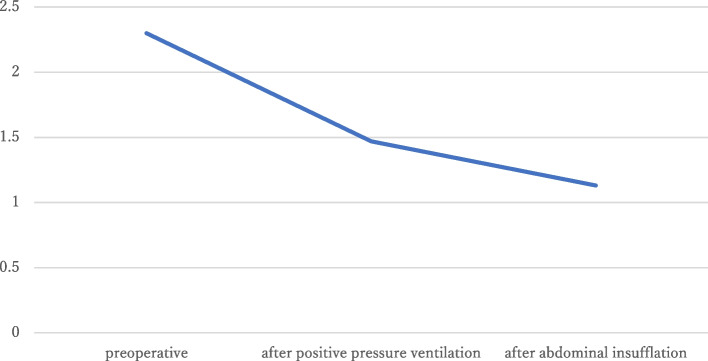
The changes of Qp/Qs. The preoperative Qp/Qs ratio was 2.3. It was reduced to 1.47 after positive pressure ventilation and 1.13 after insufflation. Qp/Qs, pulmonary to systemic blood flow ratio

A 72-year-old Japanese man: height, 159 cm; weight, 57.4 kg with bowel obstruction who underwent a laparoscopic right colectomy for ascending colon cancer was reported. The patient was diagnosed with TOF and underwent repair surgery at 19 years of age; however, he had a residual ventricular septal defect and right ventricular outflow tract obstruction. The surgical records from that time are not available and procedure details are unknown. He often experienced paroxysmal atrial flutter, which exacerbated heart failure with dyspnea, and was treated with the angiotensin-converting enzyme inhibitors spironolactone, digoxin, and flecainide for heart failure. Chest radiography revealed mild cardiomegaly, with a 50% cardiothoracic ratio; sinus arrhythmia at 93 beats per minute was observed on electrocardiography. Blood tests showed adequate hemoglobin; 15.4 g/dL, mildly elevated liver enzymes; aspartate aminotransferase, 52 U/L; alanine aminotransferase, 44 U/L, normal coagulation function, and elevated brain natriuretic peptide; 72.6 pg/ml, suggesting heart failure. Renal function was normal, with no electrolyte abnormalities. Echocardiography revealed that the diameter of the residual VSD was approximately 6 mm with left-to-right ventricular shunting; Vmax, 2.8 m/s and mild aortic and tricuspid regurgitation. Left ventricular wall motion was preserved using Simpson’s method with an ejection fraction of 57.3% (Fig. 1). The right ventricular function was slightly reduced, right ventricular fractional area change (RVFAC); 26%, tricuspid annular plane systolic excursion (TAPSE); 18 mm. Preoperative pulmonary artery catheterization revealed a pressure gradient of 57 mmHg between the right ventricle and the pulmonary artery, suggesting residual right ventricular outflow tract obstruction; HR 90 bpm, BP 105/57 mmHg, pulmonary artery pressure (PAP) 28/12 (19) mmHg, right ventricular pressure (RVP) 85/10 mmHg, right atrial pressure (RAP) 10 mmHg, pulmonary capillary wedge pressure (PCWP) 10 mmHg. The Qp/Qs was 2.3 (Fig. 2).

After inserting an epidural anesthetic catheter and establishing arterial pressure-based cardiac output (FloTrac; Edwards Life Sciences, CA, USA), general anesthesia was induced using midazolam; 3 mg, remifentanil; 0.3 μg/kg/min, rocuronium; 50 mg, following which endotracheal tube and transoesophageal echocardiography (TEE) probe were inserted. We used pressure-regulated volume control ventilation to ventilate the patient with a tidal volume of 500 mL, 10–12 breaths/min, and positive end-expiratory pressure (PEEP) of 5 cmH_2_O. Noradrenaline and dobutamine were administered continuously to avoid undesirable hypotension. Transient tachycardia with a 2:1 atrial flutter conduction ratio was observed immediately after induction, and the heart rate was controlled with landiolol. General anesthesia was maintained using desflurane. Four milliliters of 0.25% levobupivacaine, 180 ml of 0.25% levobupivacaine, and 20 ml of fentanyl were administered continuously via epidural anesthesia at 4 ml/h. The intraperitoneal pressure was maintained at 10–12 mmHg and was not changed according to pulmonary artery pressure.

Pulmonary artery catheters were inserted through the right internal jugular vein under fluoroscopic guidance. Qp/Qs was calculated using a blood gas analysis of arterial, pulmonary arterial, and mixed venous blood, assuming the oxygen saturation in the pulmonary veins to be 100%. The pressure gradient at the right ventricular outflow tract was 32 mmHg (pulmonary artery pressure, 25/17 mmHg; right ventricular pressure, 57/10 mmHg), and Qp/Qs was 1.47, which was significantly lower than those preoperative values (Fig. 2). After abdominal insufflation, the pulmonary artery pressure increased to 36/20 mmHg; however, the right ventricular pressure also increased slightly, 63/17 mmHg, resulting in a slight decrease in the pressure gradient to 27 mmHg. Qp/Qs further decreased to 1.13 (Fig. 2). The systemic vascular resistance index was 1852 dynes*sec/cm^5^/M^2^ after anesthesia induction and 1472–1714 dynes*sec/cm^5^/M^2^ after abdominal insufflation.

The dose was adjusted in the range of 0.05–0.2 μg/kg/min of noradrenaline to maintain blood pressure, 1–2 μg/kg/min of dobutamine to assist cardiac contractility, and 1–5 μg/kg/min of landiolol to control heart rate. Intraoperative hemodynamics were stable, with a cardiac index of 2.0–2.5 L/min∙m^2^ (Table 1). After surgery, the patient was extubated in the operating room. The anesthesia and operation duration were 315 and 215 min, respectively. Blood loss was 310 ml, urine output was 500 ml, infusion volume was 2250 ml, and transfusions were not needed. Continuous epidural anesthesia was administered for postoperative analgesia. The patient had a good postoperative course and was discharged from the intensive care unit on the 4th postoperative day and from the hospital on the 12th day.

**Table 1:** Hemodynamic parameters

	Preoperative	After positive pressure ventilation	After abdominal insufflation
HR (/min)	90	54	50
BP (mmHg)	105/57	76/45	74/45
PAP (mean) (mmHg)	28/12 (19)	33/17 (25)	40/22 (25)
RVP (mmHg)	85/10	57/10	63/17
CI (L/min/m^2^)	2.97	2.1	2.3
TPR (dyne/sec/cm^5^)	232	608	688
IP (mmHg)			10-12

Due to right ventricular outflow tract obstruction, the preoperative RVP was elevated, but the PAP was within the normal range. Positive pressure ventilation and insufflation increased the TPR and PAP. CI, BP, and HR were generally stable during surgery

*HR* heart rate, *BP* blood pressure, *PAP* pulmonary artery pressure, *RVP* right ventricular pressure, *CI* cardiac index, *TPR* total pulmonary resistance, *IP* insufflation pressure

## Discussion

This is the first report to measure intraoperative Qp/Qs during laparoscopic surgery in a patient with TOF and residual anomalies, including residual VSD with left-to-right shunting and pulmonary subvalvular stenosis. We preoperatively predicted that positive pressure ventilation and abdominal insufflation would increase intrathoracic pressure and pulmonary vascular resistance, decrease shunt volume, and potentially bring Qp/Qs closer to 1.0. We proved this hypothesis by measuring Qp/Qs and were able to capture the hemodynamics improvement numerically. Perioperative hemodynamics were preserved, and the patient did not develop heart failure.

Hemodynamics in ACHD are generally very sensitive to changes in preload, afterload, contractility, or arrhythmias [5]. The hemodynamic goal in the anesthetic management of adult patients with uncorrected TOF is to reduce right ventricular afterload because these patients have a certain right ventricular dysfunction due to residual pulmonary valve regurgitation or stenosis. Further, caution must be taken to avoid an imbalance between pulmonary vascular resistance and systemic vascular resistance [5]. Hemodynamic changes must be considered when selecting laparoscopic surgery. Factors modifying the hemodynamics in TOF include VSD shunt volume and direction, the ratio of pulmonary to systemic vascular resistance, pulmonary artery stenosis, and right ventricular contractility. Shunting becomes left-to-right in VSD and Qp/Qs > 1 when the pulmonary blood flow is preserved, whereas high pulmonary vascular resistance decreases Qp. In this case, right ventricular contractility and pulmonary blood flow were maintained despite long-term pulmonary subvalvular stenosis, and the VSD was a left-to-right shunt with a high shunt volume. Pulmonary vascular resistance was not high, probably because the subvalvular stenosis prevented direct stress to the peripheral pulmonary vessels. Therefore, we expected the pulmonary blood flow limitation to improve the hemodynamics in this case. The increasing intrathoracic pressure caused by mechanical ventilation and insufflation decreased the left-to-right shunt volume, bringing Qp/Qs close to 1.0. However, laparoscopic hysterectomy in adult patients with TOF and residual pulmonary atresia has been reported to increase intrathoracic pressure caused by pneumoperitoneum-induced right-to-left shunting and cyanosis with compromised pulmonary blood flow [6]. Anesthesia and surgical manipulation can alter the balance between systemic and pulmonary vascular resistance, potentially reversing the shunt.

Intraoperative TEE is useful in treating ACHD. A successful case of laparoscopic total hysterectomy in a patient with TOF and unrepaired pulmonary atresia has been reported [6], in which qualitative evaluation of the left ventricular end-diastolic volume using TEE was used as a surrogate index of left ventricular preload to prevent excessive infusion [6, 7]. It also described the advantages of TEE in terms of ventricular function over time and air embolism monitoring. In our case, TEE was useful for monitoring the shunt through the VSD and right ventricular contractility. A decrease in shunt volume was observed with artificial ventilation and abdominal insufflation using the color Doppler method. Right ventricular contractility can be monitored over time based on the rate of change in the right ventricular area.

## Conclusions

Laparoscopic surgery might be tolerable in patients with tetralogy of Fallot who have preserved the right ventricular function, left ventricular to right ventricular shunts, and do not have high pulmonary vascular resistance. Preoperative evaluation to ensure that the right ventricular function is tolerable is essential because abdominal insufflation increases the pressure load on the right ventricle. Using a pulmonary artery catheter for monitoring, hemodynamic changes can be numerically captured and managed during surgery.

## Data Availability

Data relevant to this case report are not available for public access because of patient privacy concerns but are available from the corresponding author upon reasonable request.

## Abbreviations

Qp/Qs: Pulmonary-to-systemic blood flow ratio
ACHD: Adult congenital heart disease
TOF: Tetralogy of Fallot
VSD: Ventricular septal defect
TEE: Transesophageal echocardiography
